# Optimised patient information materials and recruitment to a study of behavioural activation in older adults: an embedded study within a trial

**DOI:** 10.12688/f1000research.24051.1

**Published:** 2020-05-21

**Authors:** Peter Knapp, Simon Gilbody, Janet Holt, Ada Keding, Natasha Mitchell, David K. Raynor, Jonathan Silcock, David Torgerson

**Affiliations:** 1 Department of Health Sciences and the Hull York Medical School, University of York,, York, UK; 2 School of Healthcare, University of Leeds, Leeds, UK; 3 Department of Health Sciences, University of York, York, UK; 4 School of Pharmacy and Medical Sciences, University of Bradford, Bradford, UK

**Keywords:** SWAT, trial, recruitment, patient information, user testing, behavioral activation

## Abstract

**Background: **Printed participant information about randomised controlled trials is often long, technical and difficult to navigate. Improving information materials is possible through optimisation and user-testing, and may impact on participant understanding and rates of recruitment.

**Methods: **A study within a trial (SWAT) was undertaken within the CASPER trial. Potential CASPER participants were randomised to receive either the standard trial information or revised information that had been optimised through information design and user testing.

**Results: **A total of 11,531 patients were randomised in the SWAT. Rates of recruitment to the CASPER trial were 2.0% in the optimised information group and 1.9% in the standard information group (odds ratio 1.027; 95% CI 0.79 to 1.33; p=0.202).

**Conclusions: **Participant information that had been optimised through information design and user testing did not result in any change to rate of recruitment to the host trial.

**Registration: **ISRCTN ID ISRCTN02202951; registered on 3 June 2009.

## Introduction

Potential participants in randomised controlled trials are given information that is often long, technical and difficult to navigate ^1– 3^. Consequently, they may lack understanding of important details about the trial ^1, 4, 5^, which limits their ability to make an informed decision about consent.

Improving information materials is possible through optimisation and user-testing. This involves making changes to the design and text based on good practice in information design and people’s ability to find and understand information during testing ^6^. Materials revised after user-testing have been shown to be preferred ^7, 8^, although a recent review concluded that optimised information has little or no impact on trial recruitment ^9^. However, the evidence base remains limited ^10– 13^, and a recent ‘review of reviews’ reported that information for patients can be a facilitator of research participation ^14^.

### Study aims

This embedded study within a trial (SWAT) assesses whether optimisation of patient information materials through user testing could increase participant recruitment to the CASPER study ^15^.

## Methods

### Design

The SWAT was conducted within CASPER, which investigated the effectiveness of behavioural activation in patients aged 65 years or older with sub-threshold levels of depression ^15^. CASPER used a cohort multiple randomised controlled trial design ^16^.

### Participants

Participants were registered patients at one of six UK medical practices in Durham, Harrogate, Leeds and York. They were included if they were potentially eligible for CASPER.

### Intervention

All participants in the SWAT were posted an invitation letter, participant information sheet (PIS), screening questionnaire and consent form for the CASPER trial. The control group received the standard CASPER developed PIS (see *Extended data*) ^17^ whilst the intervention group were sent an optimised version (see *Extended data*) ^18^ developed through three rounds of user testing and revision.

Patients returned the questionnaire and a consent form indicating a willingness to participate, after which they were recruited to the CASPER cohort. Following a telephone diagnostic interview, eligible patients were recruited to the CASPER intervention trial.

### User testing

User testing involved 30 people reflecting the CASPER target population. In the first round of testing, 10 participants read the standard invitation letter and PIS. They were then asked to locate and demonstrate their understanding of 18 items of information within the PIS (on the study’s nature and purpose; process and meaning of consent; study procedures; nature of the CASPER trial intervention). The PIS was then revised based on participant responses. A second round of testing was completed, in which 10 new participants read the invitation letter and a revised PIS and were asked to find and show understanding of the same 18 information items. The PIS was further revised and tested on 10 new participants through the same 18 information items.

Through testing, changes to the PIS included adding a title page, a summary of key points and a contents page, highlighting headings using colour and larger font, and simplifying wording. The final optimised PIS was printed as an A4 booklet (Figure 3).

### Outcomes

The primary outcome measure was the proportion of patients in each group who were recruited to the CASPER trial. The secondary outcomes were (i) the proportion of patients recruited to the CASPER cohort, and (ii) the proportion of invited patients returning forms to express interest in participation in CASPER.

### Sample size

It was predicted that 30% of invited patients would return the consent form and indicate interest in CASPER participation, of whom 20% (600) would be eligible to take part in the CASPER trial. An improvement in response rate of 10% (i.e. from 30% to 33% participants) would be a significant increase in uptake. A sample size of 8,000 potential participants would be sufficient at 80% power to detect a difference of 10% in recruitment rate.

### Randomisation

Individual patients were allocated randomly (1:1) to receive either the standard or optimised PIS by an independent statistician at York Trials Unit.

### Statistical analysis

Odds ratios (ORs) and corresponding 95% confidence intervals (CIs) were calculated to compare the proportion of patients from each group that were recruited to the CASPER trial; recruited to the CASPER cohort; or expressed interest in participation. Analyses were conducted in Stata version 14.2.

### Approvals

CASPER and the SWAT were approved by the NHS Leeds North-East Research Ethics Committee (10/H1306/61).

## Results

**Figure 1.**
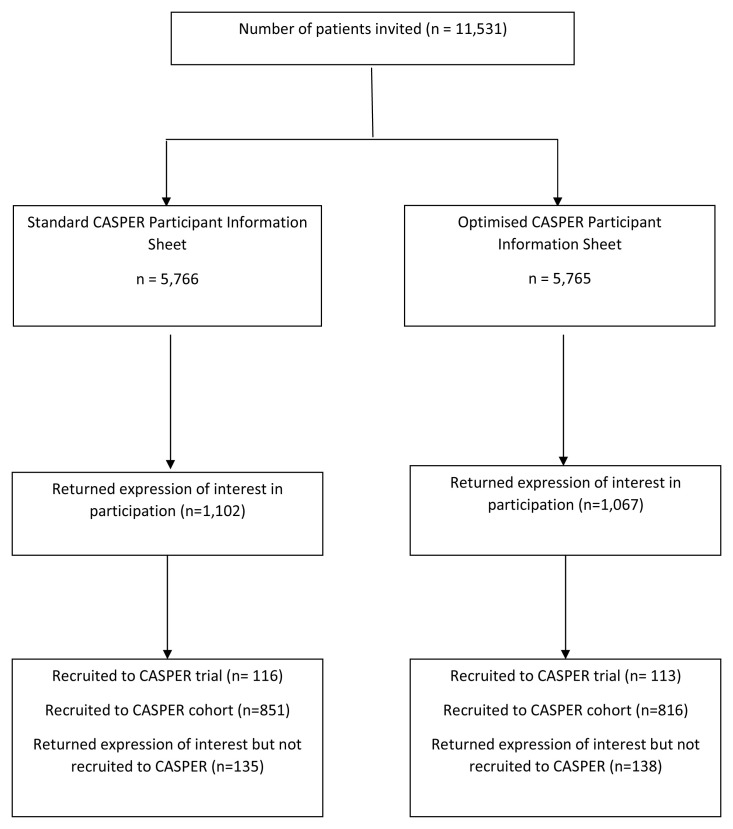
Flow diagram of recruitment to the CASPER trial.

Overall, 11,531 patients were invited to participate ^19^; 5,765 (50.0%) were randomised to the optimised PIS and 5,766 (50.0%) to the standard PIS ( Figure 1).

A total of 2,169 patients returned the consent form indicating a willingness to take part: 1,102 (19.1%) in the optimised PIS group and 1,067 (18.5%) in the standard PIS group (odds ratio (OR) 1.04; 95% confidence interval (CI) 0.95 to 1.14; p=0.402).

A total of 229 patients were recruited to the CASPER trial: 116 (2.0% of those invited) in the optimised PIS group and 113 (1.9%) in the standard PIS group (OR 1.027; 95% CI 0.79 to 1.33; p=0.202).

In total, 1,667 patients expressed interest in participating but were ineligible for the CASPER trial and were recruited to the CASPER cohort: 851 (14.8% of those invited) in the optimised PIS group, and 816 (14.1%) in the standard PIS group (OR 1.05; 95% CI 0.95 to 1.16).

## Discussion

Optimisation of the PIS resulted in no statistically significant difference in the rates of recruitment to the CASPER trial or CASPER cohort, or rates of consent form returns. This is consistent with previous research ^9^, including other embedded trials within the MRC START programme, which have observed little or no effect on recruitment ^11– 13, 20^.

Whilst there was no impact on recruitment, the optimised materials may have improved understanding of the trial thus enabling patients to make a more informed decision. Improved comprehension could also increase retention, due to greater understanding of the trial prior to recruitment. These outcomes were not assessed and further research examining this is warranted.

## Conclusion

Optimised patient information materials did not increase recruitment to the host trial or expressions of interest in participation.

## Data availability

### Underlying data

Figshare: CASPER SWAT data.csvCASPER SWAT recruitment data and evaluated information sheets. https://doi.org/10.6084/m9.figshare.12302672 ^20^.

This project contains the underlying data

### Extended data

Figshare: Figure 2 CASPER PIS (original). https://doi.org/10.6084/m9.figshare.12302675 ^17^.

This file is the original CASPER participant information sheet.

Figshare: Figure 3 CASPER PIS (revised). https://doi.org/10.6084/m9.figshare.12302678 ^18^.

This file is the revised CASPER participant information sheet.

### Reporting guidelines

Figshare: CONSORT checklist for ‘Optimised patient information materials and recruitment to a study of behavioural activation in older adults: an embedded study within a trial’. https://doi.org/10.6084/m9.figshare.12312206.v1 ^21^.

Data are available under the terms of the Creative Commons Attribution 4.0 International license (CC-BY 4.0).

## References

[1] Larson E Foe G Lally R: Reading level and length of written research consent forms. *Clin Transl Sci.* 2015;8(4):355–356. 10.1111/cts.12253 25580939 PMC5351029

[2] Manta CJ Ortiz J Moulton BW: From the Patient Perspective, Consent Forms Fall Short of Providing Information to Guide Decision Making. *J Patient Saf.* 2016. 10.1097/PTS.0000000000000310 27490160 PMC5290300

[3] Montalvo W Larson E: Participant comprehension of research for which they volunteer: a systematic review. *J Nurs Scholarsh.* 2014;46(6):423–431. 10.1111/jnu.12097 25130209

[4] Griffin JM Struve JK Collins D: Long term clinical trials: how much information do participants retain from the informed consent process?. *Contemp Clin Trials.* 2006;27(5):441–448. 10.1016/j.cct.2006.04.006 16798101

[5] Fortun P West J Chalkley L: Recall of informed consent information by healthy volunteers in clinical trials. *QJM.* 2008;101(8):625–629. 10.1093/qjmed/hcn067 18487271

[6] Raynor DK Knapp P Silcock J: "User-testing" as a method for testing the fitness-for-purpose of written medicine information. *Patient Educ Couns.* 2011;83(3):404–10. 10.1016/j.pec.2011.03.016 21530140

[7] Knapp P Raynor DK Silcock J: Performance-based readability testing of participant information for a Phase 3 IVF trial. *Trials.* 2009;10(1):79. 10.1186/1745-6215-10-79 19723335 PMC2743679

[8] Knapp P Raynor DK Silcock J: Can user testing of a clinical trial patient information sheet make it fit-for-purpose?--a randomized controlled trial. *BMC Med.* 2011;9:89. 10.1186/1741-7015-9-89 21777435 PMC3152894

[9] Treweek S Pitkethly M Cook J: Strategies to improve recruitment to randomised trials. *Cochrane Database Syst Rev.* 2018;2:Mr000013. 10.1002/14651858.MR000013.pub6 29468635 PMC7078793

[10] Chen F Rahimi K Haynes R: Investigating strategies to improve attendance at screening visits in a randomized trial. *Trials.* 2011;12(1):A111 10.1186/1745-6215-12-S1-A111

[11] Man MS Rick J Bower P: Improving recruitment to a study of telehealth management for long-term conditions in primary care: two embedded, randomised controlled trials of optimised patient information materials. *Trials.* 2015;16(1):309. 10.1186/s13063-015-0820-0 26187378 PMC4506607

[12] Cockayne S Fairhurst C Adamson J: An optimised patient information sheet did not significantly increase recruitment or retention in a falls prevention study: an embedded randomised recruitment trial. *Trials.* 2017;18(1):144. 10.1186/s13063-017-1797-7 28351376 PMC5370466

[13] Parker A Knapp P Treweek S: The effect of optimised patient information materials on recruitment in a lung cancer screening trial: an embedded randomised recruitment trial. *Trials.* 2018;19(1):503. 10.1186/s13063-018-2896-9 30227890 PMC6145341

[14] Sheridan R Martin-Kerry J Hudson J: Why do patients take part in research? An overview of systematic reviews of psychosocial barriers and facilitators. *Trials.* 2020;21:259. 10.1186/s13063-020-4197-3 32164790 PMC7069042

[15] Gilbody S Lewis H Adamson J: Effect of Collaborative Care vs Usual Care on Depressive Symptoms in Older Adults With Subthreshold Depression: The CASPER Randomized Clinical Trial. *JAMA.* 2017;317(7):728–737. 10.1001/jama.2017.0130 28241357

[16] Relton C Torgerson D O’Cathain A: Rethinking pragmatic randomised controlled trials: introducing the “cohort multiple randomised controlled trial” design. *BMJ.* 2010;340:c1066. 10.1136/bmj.c1066 20304934

[17] Knapp P Gilbody S Holt J: Figure 2 CASPER PIS (original). *figshare.*Figure.2020 10.6084/m9.figshare.12302675.v1

[18] Knapp P Gilbody S Keding A: Figure 3 CASPER PIS (revised). *figshare.*Figure.2020 10.6084/m9.figshare.12302678.v1

[19] Knapp P Gilbody G Holt J: Optimised patient information materials and recruitment to a study of behavioural activation in older adults: an embedded study within a trial (dataset). 10.6084/m9.figshare.12302672 PMC7400690 32789011

[20] Rick J Graffy J Knapp P: Systematic techniques for assisting recruitment to trials (START): study protocol for embedded, randomized controlled trials. *Trials.* 2014;15:407. 10.1186/1745-6215-15-407 25344684 PMC4230578

[21] Knapp P Gilbody S Holt J: CONSORT 2010 Checklist Knapp et al. *figshare.*Journal contribution.2020 10.6084/m9.figshare.12312206.v1

