# Supplementary material for: Optimised patient information materials and recruitment to a study of behavioural activation in older adults: an embedded study within a trial
Source: F1000Res. 2020 May 21;9:417. [Version 1] doi: 10.12688/f1000research.24051.1 (PMC7400690; doi:10.12688/f1000research.24051.1)
Supplement: Peer review history

# Peer review: Reviewer response for version 1

**DOI:** 10.5256/f1000research.26530.r67713

**Published:** 2020-08-03

**Authors:** Shaun P. Treweek

**Affiliations:** 1 Health Services Research Unit, Institute of Applied Health Sciences, School of Medicine, Medical Sciences and Nutrition, University of Aberdeen, Aberdeen, UK

This is a well-written article describing a large, well done SWAT. I only have a few comments, which are listed below.

**Study aims**

 The authors might want to add a sentence (and possibly a reference) that explains in brief what a SWAT is.

**Methods**

*Design*– linked to the host trial ‘cohort multiple randomised controlled trial design’. This design is sometimes called a ‘Trials Within Cohort (TwiCs)' design. The authors might want to mention this name too, just in case readers are more familiar with it.  

*User testing*– the authors mention users were asked to locate and demonstrate understanding of 18 items of information within the participant information leaflet. I wasn’t sure what the 18 items were. Would it be possible to list them? Apologies if I have missed them within the article or extended data.

*Randomisation*– could the authors give a little more information on how the randomisation sequence was generated?

**Discussion**

 The authors mention that their results are consistent with previous research, which is my understanding of the literature too. Do the authors think further evaluation of this SWAT evaluation is required, and if so, could they give some pointers to what sorts of evaluations would add most value to the evidence base?

Is the work clearly and accurately presented and does it cite the current literature?

Yes

If applicable, is the statistical analysis and its interpretation appropriate?

Yes

Are all the source data underlying the results available to ensure full reproducibility?

Yes

Is the study design appropriate and is the work technically sound?

Yes

Are the conclusions drawn adequately supported by the results?

Yes

Are sufficient details of methods and analysis provided to allow replication by others?

Yes

Reviewer Expertise:

Randomised trial methodology, including SWATs.

I confirm that I have read this submission and believe that I have an appropriate level of expertise to confirm that it is of an acceptable scientific standard.

# Peer review: Reviewer response for version 1

**DOI:** 10.5256/f1000research.26530.r67160

**Published:** 2020-07-30

**Authors:** Mike J. Clarke

**Affiliations:** 1 Centre for Public Health, School of Medicine, Dentistry and Biomedical Sciences, Institute of Clinical Sciences, Block B, Royal Hospital, Queen's University Belfast, Belfast, UK

This is an impressive and important piece of methodology research, which has been reported in a clear and succinct manner. I am pleased to have been asked to review it.

 The reported randomized trial is an example of a very large SWAT, that was embedded in the CASPER trial of behavioural activation therapy for older patients with sub-threshold levels of depression, to test the impact on recruitment of an optimized information materials for people being invited to consider participation in the clinical trial.

 It might be helpful to readers if the authors could include a little more information about the SWAT concept, perhaps by citing one or both of the relevant Trial Forge papers: (a) Treweek S, Bevan S, Bower P, et al. Trial Forge Guidance 1: what is a Study Within A Trial (SWAT)? Trials 2018; 19: 139; and (b) Treweek S, Bevan S, Bower P, et al. Trial Forge Guidance 2: how to decide if a further Study Within A Trial (SWAT) is needed. Trials 2020; 21: 33.

 Although the optimized information materials did not improve the proportion of invitees who were recruited into CASPER, it would be interesting to know if there were other benefits, such as a better understanding of the clinical trial. The paper concludes with some comments about this but the authors' comment that “further research examining this is warranted” seems to suggest that this type of qualitative research might not be done with the CASPER trial patients. However, given that they will be collecting retention data for CASPER, I hope they will revisit this SWAT later in the CASPER trial to answer their own question about the potential for an impact on retention.

 If they have not already done so, I suggest that the authors register this SWAT in the SWAT repository. It might also help readers if they referred to two similar SWAT that are already registered there (SWAT 101 and SWAT 105).

Is the work clearly and accurately presented and does it cite the current literature?

Yes

If applicable, is the statistical analysis and its interpretation appropriate?

Yes

Are all the source data underlying the results available to ensure full reproducibility?

Yes

Is the study design appropriate and is the work technically sound?

Yes

Are the conclusions drawn adequately supported by the results?

Yes

Are sufficient details of methods and analysis provided to allow replication by others?

Yes

Reviewer Expertise:

Health services research. Randomized trials. Systematic reviews. Methodology research.

I confirm that I have read this submission and believe that I have an appropriate level of expertise to confirm that it is of an acceptable scientific standard.
